# Supplementary material for: Engineering of frustration in colloidal artificial ices realized on microfeatured grooved lattices
Source: Nat Commun. 2016 Feb 1;7:10575. doi: 10.1038/ncomms10575 (PMC4740443; doi:10.1038/ncomms10575)
Supplement: Supplementary Information — Supplementary Figures 1-7, Supplementary Notes 1-3 and Supplementary References [file ncomms10575-s1.pdf]

Supplementary Information

Supplementary figures

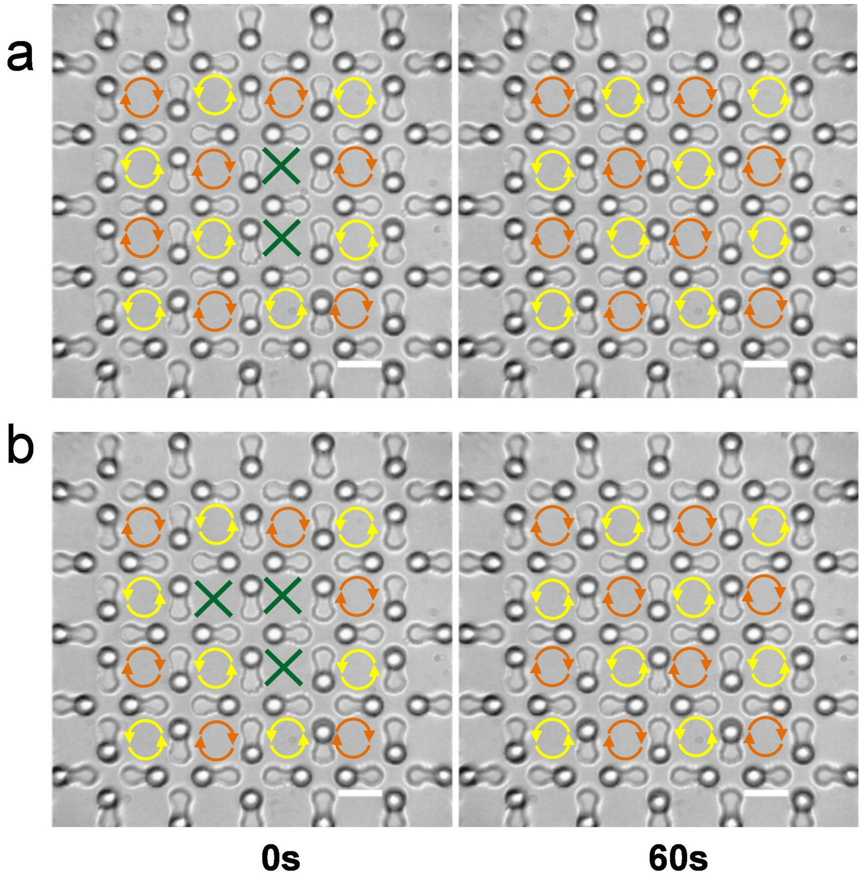

Supplementary figure 1. Unstable achiral cell in square lattice. (a,b) Microscope images showing the evolution with time of two (a) and three (b) non chiral cells in a square lattice when subjected to a static field of B = 25 mT after 60s. Scale bars are 20 μm for all images.

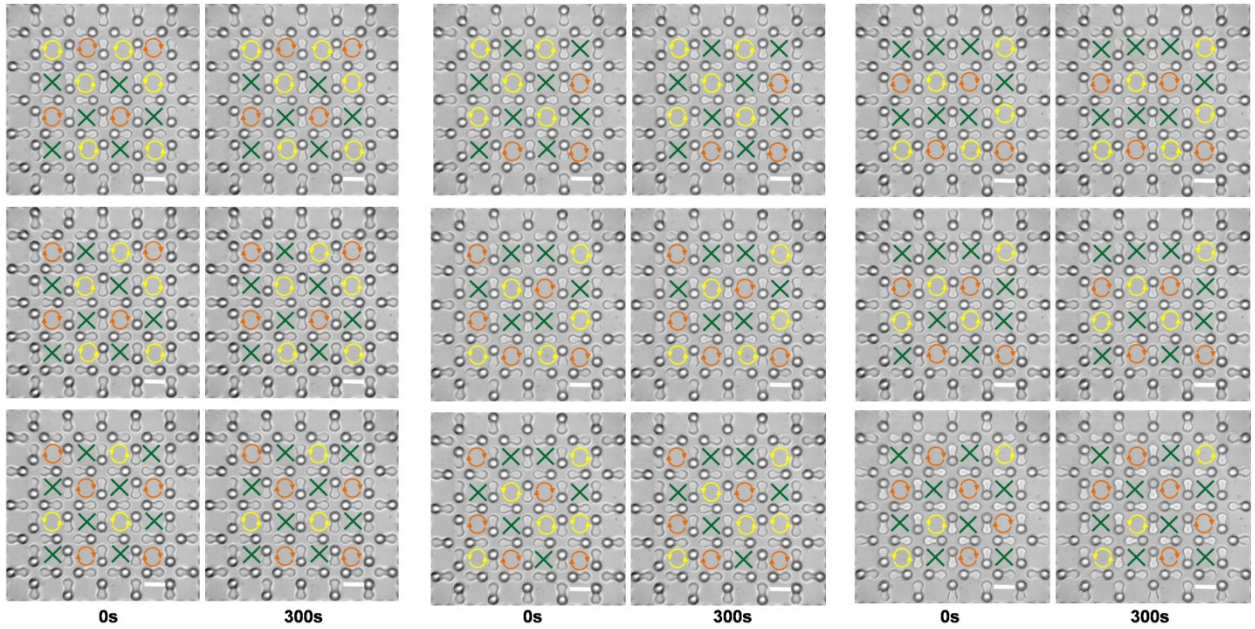

Supplementary figure 2. Stable achiral cell in square lattice. Series of microscope images showing the evolution with time of different non chiral cells in a square lattice when subjected to a static field of B=25 mT. the image is organize in three column and three rows containing in total 9 different experiments. Each experiments show the initial state (t=0) and the corresponding final unchanged state after 300s. Scale bars are 20 μm for all images.

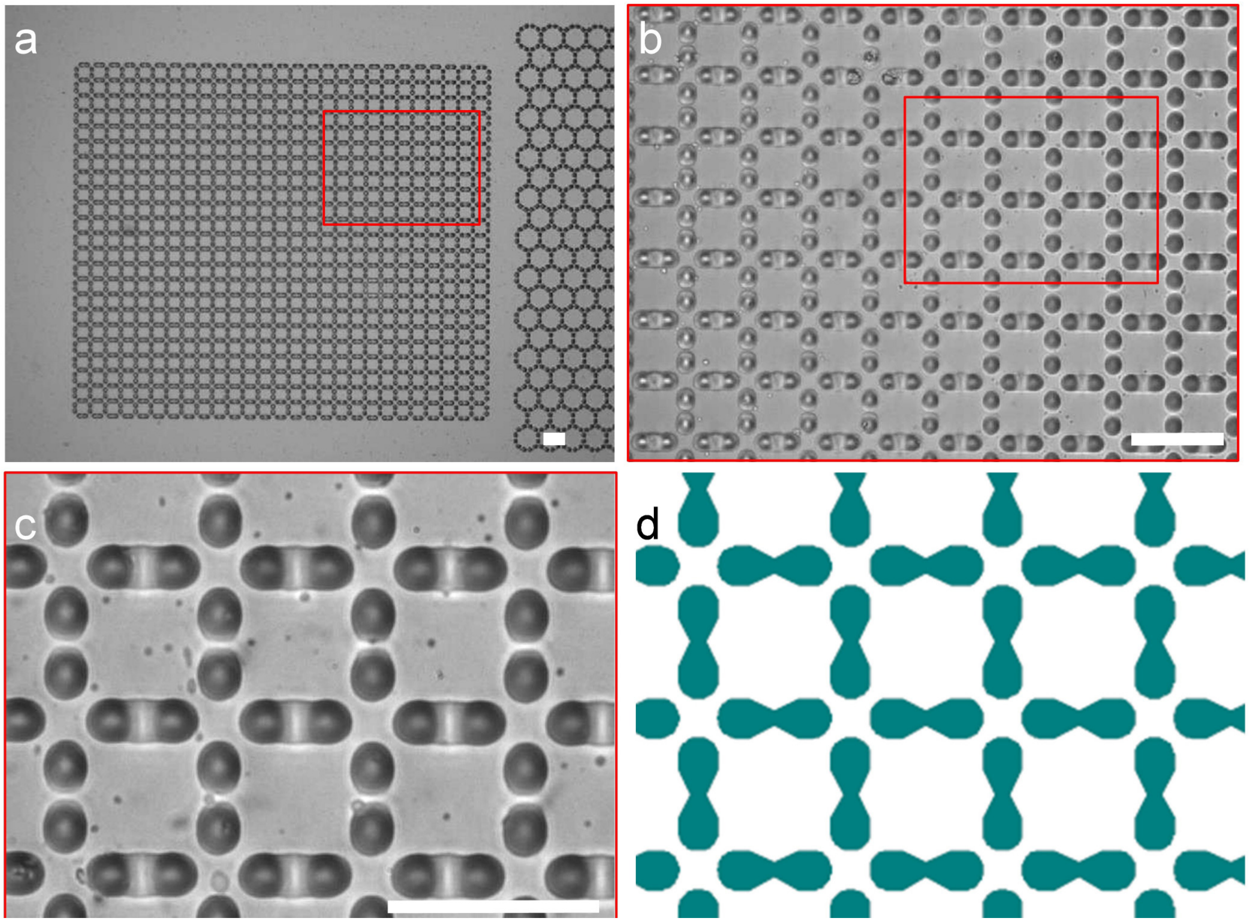

Supplementary figure 3. **Square lattice of double wells.** (a-c) Optical microscope images showing different portions of a photoligraphic square lattice of double wells islands. Scale bars for all images are 40μm. (d) Sketch of the designed lattice corresponding to the images in (d) showing the narrow constrictions present in each island to create the central hill. Scale bars are 50 μm for all images.

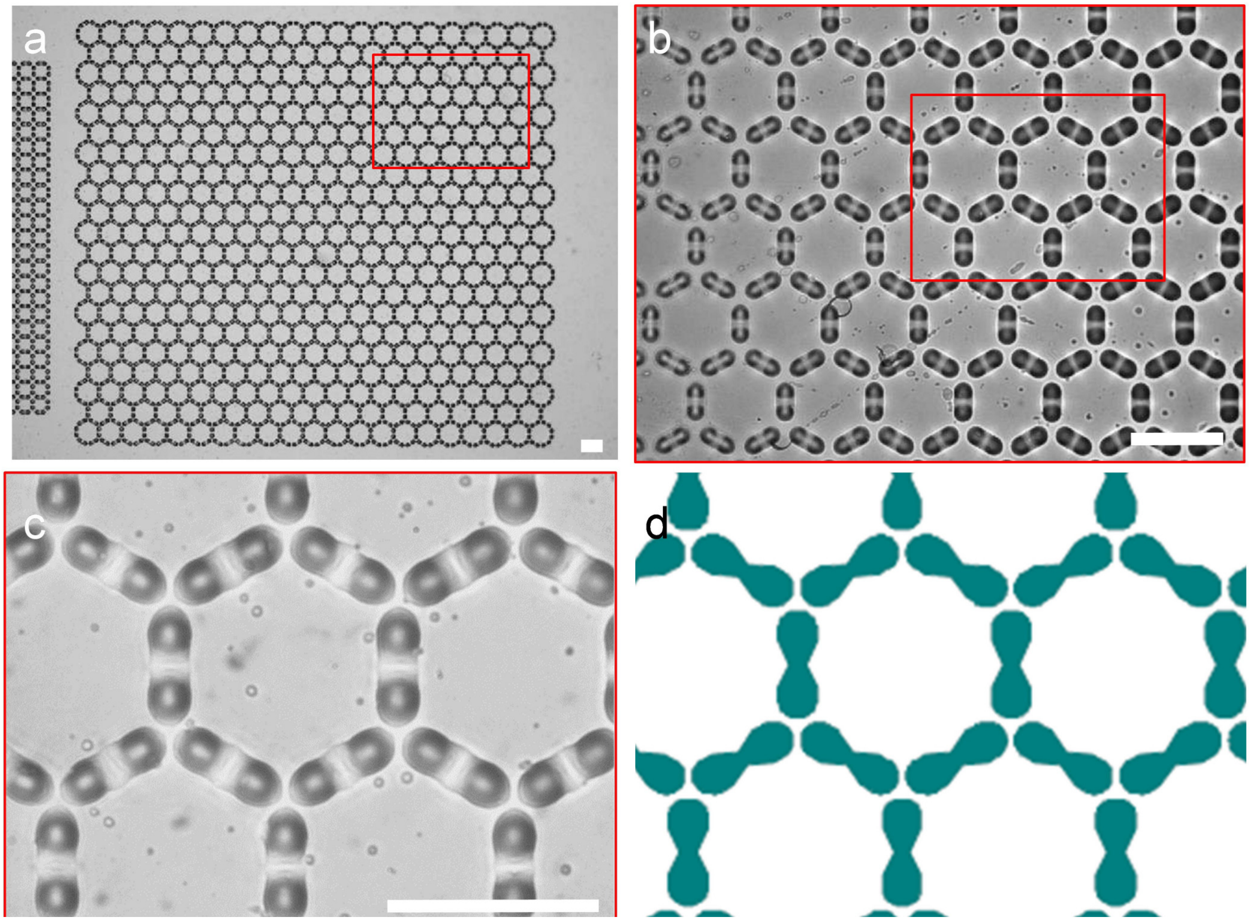

Supplementary figure 4. Honeycomb lattice of double wells. (a-c) Optical microscope images showing different portions of a soft-lithographic honeycomb lattice of double wells islands. Scale bars for all images are μm. (d) Sketch of the designed lattice corresponding to the images in (d) showing the narrow constrictions present in each island to create the central hill. Scale bars are 50 μm for all images.

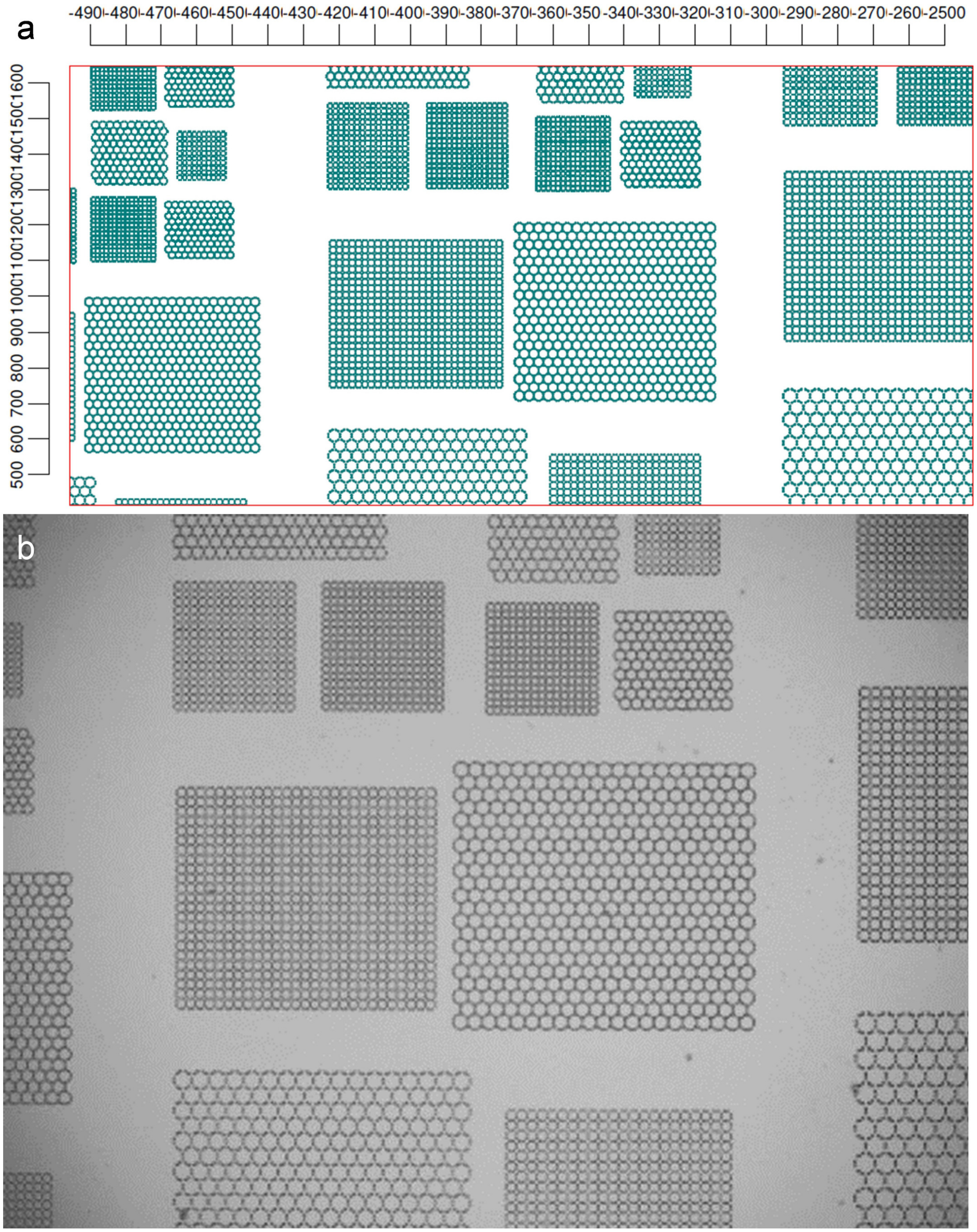

Supplementary figure 5. Comparison between designed and fabricated structures. (a) Large overview of a series of structures designed with the CleWin v.5 (PhoeniX Software, 2002). (b) Corresponding optical microscope images of these structures after the soft lithography process.

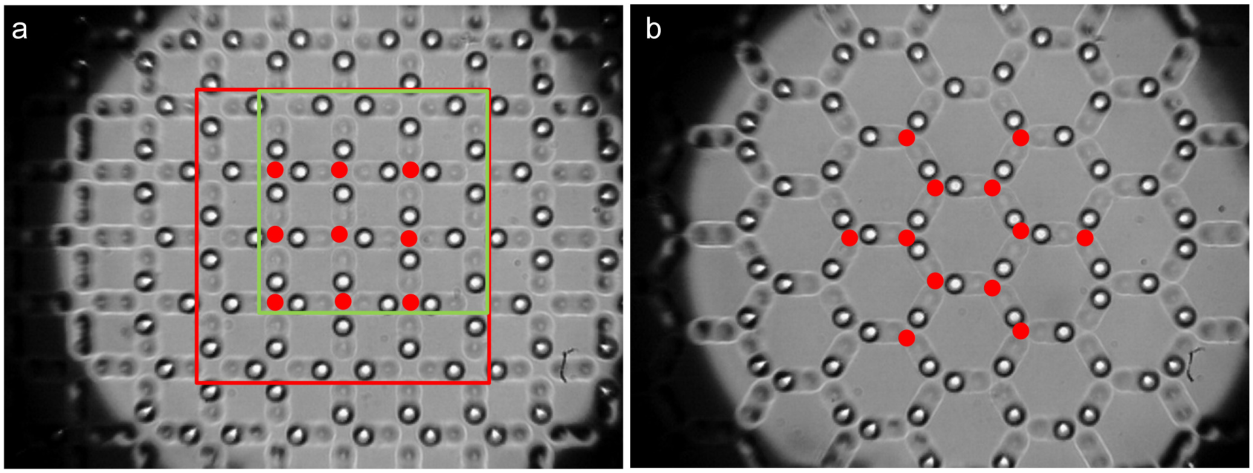

Supplementary figure 6. Boundary conditions. (a,b) Large overview of the square (a) and kagome (b) lattices filled with paramagnetic colloids. The red circles denotes in both cases the vertices considered in Figure 2a of the main article. The green rectangle in (a) correspond to the area considered by Libal et al.[2] while the red rectangle represents the area considered in our experimental system.

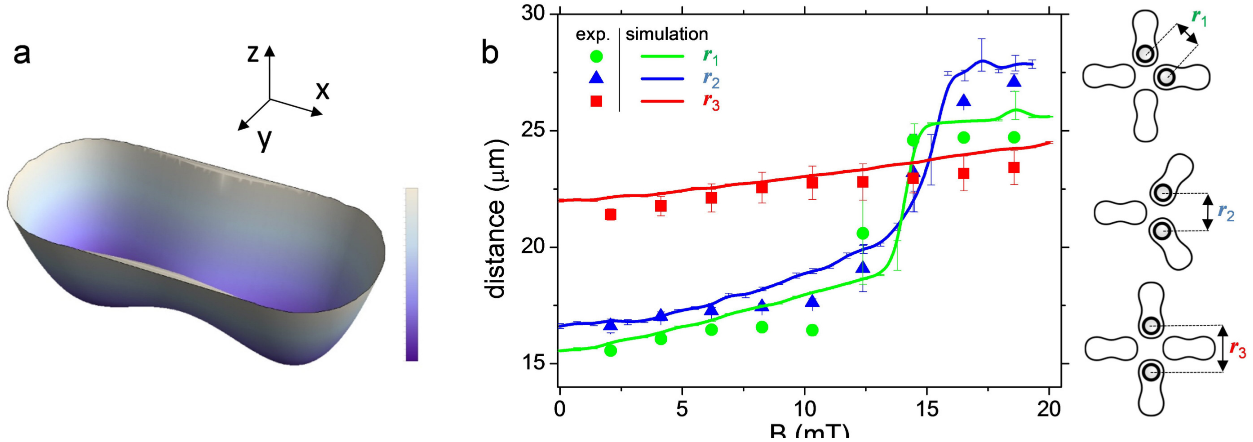

Supplementary figure 7. Double well potential and dynamics of isolated pair of particles. (a) 3D colormap of the double-well potential used in the simulations. Blue (white) region denote energy minima (maxima). (b) Evolution of the average separation distance between isolated pair of particles placed in a square (r_1 and r_3) and kagome (r_2) vertex of lattice unit cell. Scattered points represent experimental data while continuous lines result from numerical simulation. The points are the result of ten repeats, the error bars show the standard deviation of the repeat.

Supplementary notes

Supplementary note 1: Stability and evolution of chiral and achiral cells. In Supplementary figure 1 and Supplementary figure 2 we show different types of structures containing chiral and achiral cells inside a square lattice. In each of these structures the initial configuration was the ground state where every vertex is in the two in two out (S_{III}) configuration. Defects were introduced by flipping one or more particles. The particle flipping process was done by displacing one particle from its well to the nearest one using the laser tweezers. Once created, the defects are annihilated by applying the external field. Supplementary figure 1 shows unstable configurations which can easily be reduced to the ground state after application of an external field having amplitude B=25 mT. In particular, Supplementary figure 1a shows a pair of defects written by flipping one spin, which creates two achiral cells within a lattice of chiral ones. Supplementary figure 1b shows three achiral cells obtained by flipping two spins. In contrast in Supplementary figure 2 we show different combinations of a-chiral cells which were found stable for all the duration of the experiments under the maximum applied field of B = 25 mT.

Supplementary note2: Surface functionalization. During the first experimental tests we found that after sedimentation above the lithographic structure, some particles were irreversibly stuck to the surface. To avoid this problem, we give a net charge to the resin by coating it with a thin layer of polysodium 4-styrene sulfonate by using the layer-by-layer adsorption technique[1]. We prepare two stock solutions containing, respectively, 0.375g of polyallilamide hydrochloride (PAH Sigma Aldrich), in 75ml of high deionized water (MilliQ system, Millipoire) and the second with 0.375g of polysodium styrene sulfonate (PSS Sigma Aldrich) in 75ml of high deionized water. We added 2.19g of NaCl to both solutions to facilitate the adhesion of the polymer to the photoresist. The structures are submerged in the PAH solution for 30 min, then washed with high deionized water before submerging in the PSS solution for 30 min. This procedure is repeated three times before the experiments. The PAH is a strong positively charged polyelectrolyte while the PSS a negative one. After the procedure we obtain a negative charge multilayer above the photoresist capable of avoiding particle sticking. This effect results from the electrostatic interaction of the particles with the substrate, where the particles are negatively charged due to dissociation of surface COOH groups.

Supplementary note3: Periodic boundary conditions. The implementation of perfect periodic boundary conditions as done in the numerical work of Libal et al.[2] is an idealized situation, not

possible with a real experimental set-up. However in the same article the authors showed that a system with as few as 24 traps and open boundary conditions still exhibits an ice-like behavior. In order to avoid boundary effects in the statistics of the vertex counting (Figure 2(a) of the main article), we use a larger number of particles and count only the vertices surrounded by a two particle corona. Supplementary figure 6a and 6b show the considered vertices as red disks within the square (Supplementary figure 6a) and honeycomb (Supplementary figure 6b) lattice. In Supplementary figure 6a we compare the lattice used by Libal et al.[2] showed inside a green rectangle, with our experimental area inside a red square. The results reported in Fig.2(a) of the main articles are average from 10 different experiments for each field used.

Supplementary references

1. Decher, G. Fuzzy Nanoassemblies: Toward Layered Polymeric Multicomposites. *Science* **277**, 1232 (1997).
2. Libál, A., Reichhardt, C. & Reichhardt, C. J. O. Realizing Colloidal Artificial Ice on Arrays of Optical Traps. *Phys. Rev. Lett.***97**, 228302 (2006).
